# Public private partnership in the training of doctors after the 1990s’ health sector reforms: the case of Tanzania

**DOI:** 10.1186/s12960-019-0372-6

**Published:** 2019-05-22

**Authors:** Nathanael Sirili, Gasto Frumence, Angwara Kiwara, Mughwira Mwangu, Isabel Goicolea, Anna-Karin Hurtig

**Affiliations:** 1 Department of Epidemiology and Global Health, Umeå University, 90185 Umeå, SE Sweden; 2 Department of Development Studies, School of Public Health and Social Sciences, Muhimbili University of Health and Allied Sciences, P.O.BOX 65454, Dar es Salaam, Tanzania

**Keywords:** Health sector reforms, Training of doctors, Policy analysis, Public private partnership, Tanzania, Health workforce shortage

## Abstract

Similar to many other low- and middle-income countries, public private partnership (PPP) in the training of the health workforce has been emphasized since the launch of the 1990s’ health sector reforms in Tanzania. PPP in training aims to contribute to addressing the critical shortage of health workforce in these countries. This study aimed to analyse the policy process and experienced outcomes of PPP for the training of doctors in Tanzania two decades after the 1990s’ health sector reforms. We reviewed documents and interviewed key informants to collect data from training institutions and umbrella organizations that train and employ doctors in both the public and private sectors. We adopted a hybrid thematic approach to analyse the data while guided by the policy analysis framework by Gagnon and Labonté. PPP in training has contributed significantly to the increasing number of graduating doctors in Tanzania. In tandem, undermining of universities’ autonomy and the massive enrolment of medical students unfavourably affect the quality of graduating doctors. Although PPP has proven successful in increasing the number of doctors graduating, unemployment of the graduates and lack of database to inform the training needs and capacity to absorb the graduates have left the country with a health workforce shortage and maldistribution at service delivery points, just as before the introduction of the PPP. This study recommends that Tanzania revisit its PPP approach to ensure the health workforce crisis is addressed in its totality. A comprehensive plan is needed to address issues of training within the framework of PPP by engaging all stakeholders in training and deployment starting from the planning of the number of medical students, and when and how they will be trained while taking into account the quality of the training.

## Background

From the mid-1980s to the late 1990s, many low- and middle-income countries (LMICs), including Tanzania, launched health sector reforms in response to the urging of the World Bank (WB) and International Monetary Fund (IMF) as a means to improve deteriorating health systems [1]. The reforms intended for governments in LMICs to adopt structural adjustment programmes (SAPs) and thus allow market forces to determine the production and allocation of health care resources [2].

The reforms called for a sector-wide approach that needed more resources that were not readily available from many state governments following the major economic depression of the 1970s to 1980s [1, 3]. Therefore, the World Health Assembly called on the World Health Organization (WHO) to mobilize and encourage partnerships between state and non-state actors in health systems of many countries in the form of public private partnership (PPP) [4]. PPP is defined as a collaboration between public and private sector organizations where there is a pooling together of resources (financial, human, technical, and information) from public and private sources to achieve a commonly agreed social goal [5].

Although many low- and middle-income countries adopted PPP unwillingly, it was viewed as being necessary due to the economic turbulence that faced many countries in the 1980s [1, 6]. By the mid-1980s, most of the governments in many countries had failed to adequately train, employ, or retain enough doctors in their health systems [7]. The latter issue saw some doctors leaving their countries in search of greener pastures in higher-income countries [8–10].

In Tanzania, the introduction of PPP was preceded by amendment of the private hospital regulation act of 1977 that banned the private practice in health sector in the country [11]. The introduction of PPP paved the way for opening up of private health training institutions which trained different health professions including medical doctors in Tanzania. The training of the medical doctors, which had been solely the responsibility of the government, was now a subject of both the government and the private sector [12]. To embrace the concept of PPP, the government of Tanzania provided sponsorship in the form of grants to medical students even those registered in private medical training institutions [13]. Through PPP, the government was supposed to regulate and control quality, quantity, and cost of training which, if otherwise left to market forces, could have unbearably negative outcomes to both the graduates and the country [14].

However, about 20 years later, since the introduction of PPP in the training of doctors, Tanzania like for many low- and middle-income countries still suffer from a critical shortage of doctors. Taking into account the growing population, the situation has remained almost the same as before the reforms [15, 16]. This study therefore aimed to analyse the policy process and experienced outcomes of public private partnership in the training of doctors in Tanzania two decades after the 1990s’ health sector reforms.

### Conceptual framework

**Fig. 1:**
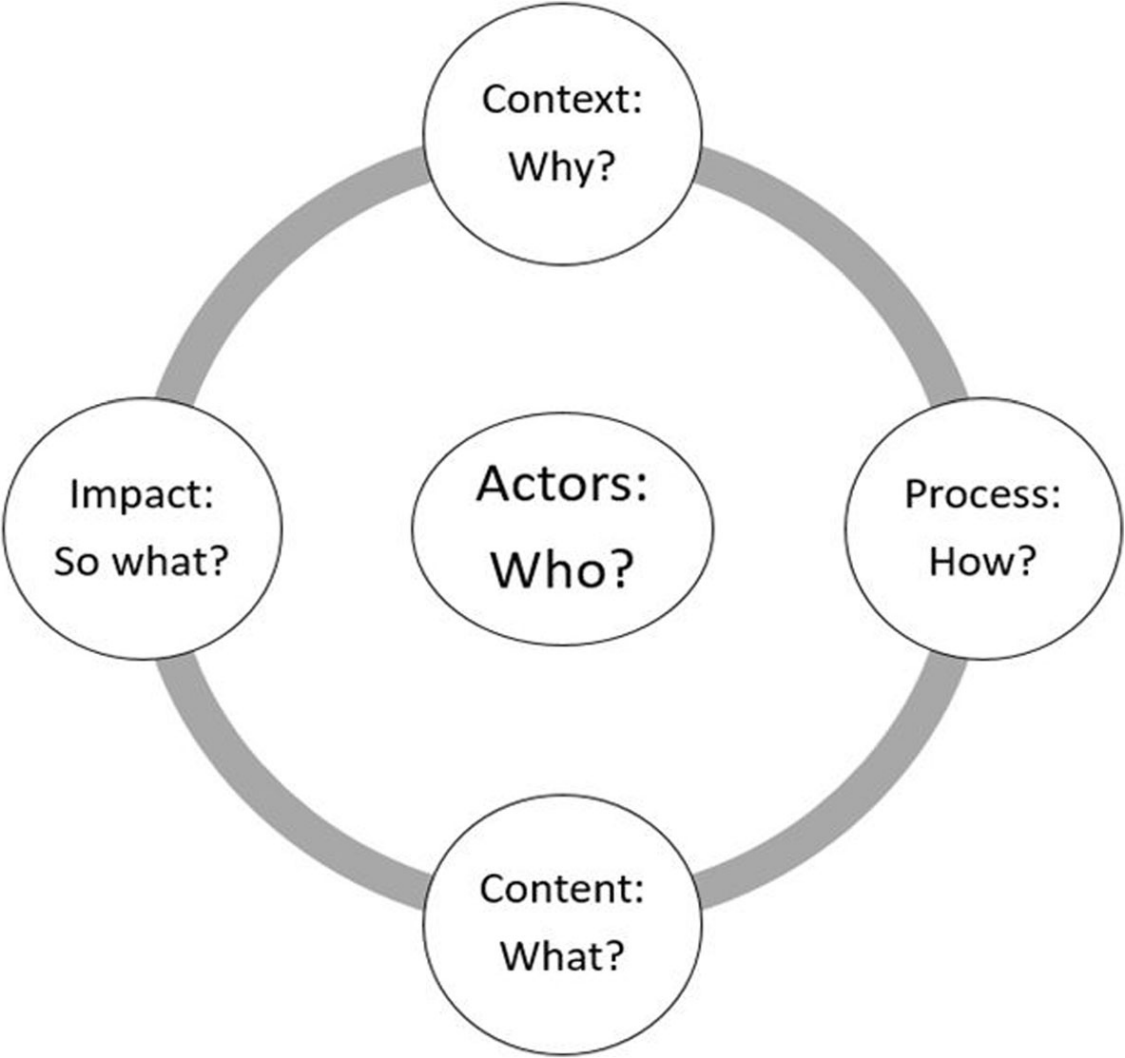
Policy analysis framework by Gagnon and Labonté

We adopted the policy analysis framework by Gagnon and Labonté (Fig. 1) to guide policy analysis on PPP in the training of doctors after the 1990s’ health sector reforms in Tanzania [17]. The framework is a modified Walt and Gilson policy analysis triangle [18]. This framework recognizes that policy is a product of five interrelated elements. These are (i) the context within which the policy is formulated and implemented; (ii) the actors involved in policy formulation and execution; (iii) the process, how the policy was formulated and is implemented; (iv) the content of the policy, i.e. what the policy intends to achieve and how such achievements will be realized; and (v) the impact, i.e. both the potential and actual effects of the policy.

We used this framework as a conceptual tool to prompt and organize the different elements (context, actors, content, process, and impacts) and their interactions in the training of doctors under the PPP era after the 1990s’ health sector reforms [19]. We assumed that the context of a health workforce crisis in a turbulent economic situation and pressure from the IMF and WB influenced the formulation and implementation of PPP in the training of doctors in Tanzania. The actors, behaviour, values, and understanding of the health workforce crisis in the country influence the implementation process and impacts of PPP policy. The contents of PPP policy for the training of doctors is not divorced from the actual and perceived powers of the actors that are involved in the formulation and implementation of it [18, 19]. The impact of a policy is a result of the interactions of all other elements in the framework. In this analysis, the impacts of the policy are limited to outcomes of the policy.

## Methods

### Study setting

This study was carried out in the Tanzanian mainland between January and May 2017. The Tanzanian mainland is one of two parts which form the United Republic of Tanzania; the other is Zanzibar. Tanzania is within the East African Great Lakes region. The Tanzanian mainland—then, Tanganyika—acquired its independence from the British in 1961 and, in 1964, united with Zanzibar to form the United Republic of Tanzania. The total population of the Tanzanian mainland is currently estimated at 47 million people [20].

The Tanzanian mainland operates a mixed health system, where both the government and the private sector have been involved in the training of the health workforce and provision of health care services since the 1990s’ health sector reforms. The private health sector is comprised of private for-profit and private not-for-profit (which forms the larger part of the private sector) [21].

The 5-year medical training for the award of the degree of Doctor of Medicine (MD) is acquired through either direct entry from high school or indirect entry through an advanced diploma or a diploma in clinical medicine. A complete high school education in Tanzania is a total of 13 years, comprised of 7 years in primary school, 4 years in ordinary level secondary school, and 2 years of advanced secondary school. After successful completion of the 5-year medical training, the graduates are subjected to a mandatory 1-year internship programme which upon successful completion they are allowed to register for the medical practice. After registration, one may opt for employment in the public or the private sector; however, the private sector is still at its infancy in Tanzania, and thus, the main viable option for employment in Tanzania is the public sector.

### Study design

We adopted a qualitative case study; this approach enables one to enquire about a contemporary phenomenon within its real-life context [22]. This design was used because the training of doctors under PPP is complex and context-dependent and involves social processes. We limited our case study to PPP in the training of doctors after the 1990s’ health sector reforms.

#### Document review

We reviewed both published and grey literature to understand the context for the introduction of PPP, the content of PPP in training, actors involved, and the implementation process of PPP in the training of doctors in Tanzania (Table 1).

**Table 1:** Data sources

Source of data	Data type and volume	Area assessed in the cycle
Documents	▪ Published reports and articles ▪ The Private Hospitals (Regulation Amendment) Act, 1991 ▪ The proposal for health sector reforms, 1994 ▪ National Higher Education Policy, 1999 ▪ The Universities Act, 2005 ▪ The National Public Private Partnership (PPP) Policy, 2009	▪ Content ▪ Context ▪ Actors ▪ Process ▪ Impact (outcomes)
Key informants	▪ Twelve interviews with key informants from the public sector (four government ministries and one training institution) ○ Ministry of Health, Community Development, Gender, Elderly and Children (MoHCDGEC) ○ President’s Office, Regional administration and local Government (PORALG) ○ Ministry of Education, Science and Technology (MEST) ○ President’s Office Public Service Management (POPSM) ○ Muhimbili University of Health and Allied Sciences (MUHAS) ▪ Eight interviews with key informants from the private sector ○ Four interviews with key informants from private not-for-profit training institutions ○ Two interviews with key informants from the private for-profit training institution ○ One interview with a key informant from the Christian Social Services Commission ○ One interview with a key informant from the Association of Private Health Facilities in Tanzania	▪ Process ▪ Impact (outcomes)

#### Key informant interviews

We used key-informant interviews to gather information on the process and perceived outcomes (impacts) of the reforms, whereby we focused our analysis on the outcomes of PPP on the training of doctors (Table 1). This study used purposive and chain referral sampling to reach key informants from government ministries, training institutions in both the public and private sectors, and informants from two umbrella associations in the employment of doctors: the Association of Private Health Facilities in Tanzania (APHFTA; private for- profit) and the Christian Social Services Commission (CSSC; private not-for-profit).

Twelve key informants came from the public institutions (10 from four ministries and two from the medical school) and the rest came from the private sector (six from medical schools and two from employers).

We developed a semi-structured interview guide to conduct the key-informant interviews based on the policy analysis framework by Gagnon and Labonté (Fig. 1) as informed by the findings from our document review [17]. Our interview guide contained questions that focused on the implementation of PPP and its impact on the training of doctors in Tanzania. Consistent with qualitative research methods, the interviewer maintained an open stance, probing into emerging themes and seeking clarification when necessary to ensure the generation of rich information from the informants. The first author carried out all interviews in the Swahili language in the office of each informant and audio-recorded the interviews using a digital audio recorder. During the interview process, the research assistant who accompanied the first author took field notes. Each interview lasted between 40 min and 1 h and 35 min. We stopped the data collection after the 20th interview once information saturation had been attained. The age of all participants ranged between 38 and 63 years.

### Data analysis

First, we transcribed verbatim all audio-recorded interviews and then translated them from Swahili to English. In order to become familiar with the content and context, all authors read the full transcripts and field notes repeatedly before the start of analysis.

We used a hybrid thematic data analysis approach; this approach used both inductive and deductive reasoning [23]. We developed an initial codebook for data analysis, based on our study objective and the policy analysis framework by Gagnon and Labonté. We then refined the codebook from the themes which emerged during the analysis. The inductive and deductive approach helped us to appropriately capture the impacts of PPP in the training of doctors in Tanzania while at the same time focusing our analysis [23]. The first author developed the initial codebook and shared it with all authors. The codebook was discussed and further developed, and a final codebook was imported into NVivo 11 qualitative data analysis computer software. The agreed codebook was tested by coding the first two interview transcripts; three authors carried this out. Their coding was almost similar, and hence, the codebook was not modified at this time.

We coded the meaningful units of text to the codes (nodes) that were found to represent that unit. Some of the meaningful units were coded more than once. At this stage, although the data analysis was guided, it was not confined to the primary codes. Inductive coding was assigned to text segments which represented a new theme that was not pre-determined. The new codes were assigned as separate codes or an expansion of the codes available in the initial codebook. Through comparisons for checking similarities and differences, the codes were sorted into categories that were further aligned into themes. Although the whole process of analysis was iterative, at this stage, further scrutiny was carried out by going back to the interview transcripts and the documents reviewed to identify, summarize, and retain the patterns and similarities, differences, and newly emerged themes. Finally, we further clustered the sub-themes and the thematic components of our study framework and presented them with supporting and succinct quotes which describe the meaning underpinning each theme.

### Ethical considerations

Muhimbili University of Health and Allied Sciences granted ethical clearance for this study (Ref. No. 2016-12-23/AEC/Vol. XII/07). We sought permission to conduct the study from the permanent secretaries of the ministries and heads of the institutions involved in data collection. We obtained written informed consent from each informant before commencing the interview.

## Results

The result section is organized into three thematic areas. We first present the findings on the context and actors; this is followed by our findings about the content and process. We conclude the results’ section by presenting the results of implementation of PPP and its perceived outcomes on the training of doctors from key informant interviews; these are complemented by quotations from the document review.

### Context and actors

Before the 1990s’ health sector reforms, the government was solely responsible for the training of doctors in Tanzania. Following the global economic crisis of the 1980s, economic hiccups resulting from the break-up of the East African community in 1977, resource diversion to support the Uganda–Tanzania War in 1978/1979, and the reduction of donor support, the government capacity to fund the health sector declined substantially [24]. With the worsening of economic conditions in Tanzania, the IMF and WB advised the country to adopt SAP and thus allow market forces to determine the uptake and allocation of health care [1]. Adoption of SAP had substantial effects on Tanzania’s communitarian ideology. The government of Tanzania suggested a dialogue about this and a stepwise adoption of SAP [24]. The steps included starting with an economic recovery programme called the National Economic Survival Programme (NESP) [24]. However, NESP was short-lived and economic conditions did not improve as fast as would have been desirable. In addition, development partner support declined given the worldwide financial crisis [24]. By the mid-1980s, the country had to adopt the major social and economic reforms due to the worsening economic situations [24, 25]. Among others, it reduced its spending on social services, including health [25]. As a result, funding in the health sector fell to below 50% of previous budgets and the share of health in the national budget fell from 30% in 1984 to 5% in the early 1990s [26, 27]. With the limited resources, reduction of spending in social services declined in all aspects, including the training of doctors. The government’s capacity to supply a sufficient health workforce, including the training and employment of the few doctors it produced, declined [26, 27].

In order to train doctors, the government called for private individuals and organizations to establish medical schools. The government felt that pooling together human and non-human resources from both the public and private sectors was a viable option to try to rescue the deteriorating health sector [24]. The government, through the ministry responsible for higher education and its units in collaboration with ministries responsible for health, local government, and finance, called for partnership with the private sector (Table 2). The public- owned medical universities are semi-autonomous in the sense that they plan and execute their roles as stated in their charters while receiving financial support from the government [28]. The private-owned medical universities fall into two categories: private for-profit universities and private not-for-profit universities. Currently, the available private not-for-profit universities are under the ownership of faith-based organizations.

**Table 2:** Actors and their roles in the training of doctors in Tanzania

Actor	Role(s)
Ministry of Higher Education, Science and Technology (MHEST)	▪ Oversees and coordinates the training of medical doctors ▪ Regulates the medical training at the universities ▪ Participates in curriculum development for medical training ▪ Provides loans and grants to medical students ▪ Pays tuition fees to selected students in both the public and private universities ▪ Provides subsidies to public universities
Ministry of Health (MoH)	▪ Oversees and coordinate internship programme ▪ Participates in curriculum development for medical training
Ministry of Finance (MoF)	▪ Overall financer of the government budget in the public sector ▪ Responsible for the budget for loans and grants
Prime Minister’s Office Regional Administration and Local Government (PMORALG)	▪ Grants permission and support to lower cadres (Clinical Officers, Assistant Medical Officers) who want to join medical schools ▪ Facilitates and coordinate the use of public health facilities for training by both the public and private institutions at the regional, municipal, and district levels
Public-owned Medical Universities	▪ Selects and admits medical students ▪ Trains medical students ▪ In collaboration with MHEST and MoH prepares and reviews curriculum for medical training
Private-owned Medical Universities	▪ Selects and admits medical students ▪ Trains medical students ▪ In collaboration with MHEST and MoH prepares and reviews curriculum for medical training

### Content and process

PPP in the training of doctors aimed to pool together human and non-human resources from both the public and private sectors and to ensure training of an adequate number of doctors in order to curb the critical shortage of doctors in points of services delivery in the country [29]. To adopt PPP, the 1977 private hospital services regulation act that banned commercialization of health services was amended in 1991. The amendment of this act allowed private individuals and organization to open and own private health training institutions [11]. To guide the implementation of PPP, a strategy note was prepared in 1993 and, in 1994, the government came up with the proposal for health sector reforms [26]. The proposal highlighted the need for a skilled health workforce at all levels of health care system and therefore proposed that health workforce development and deployment be prioritized. It also stressed that the role of the private sector should be realized through the introduction of a public private mix (currently known as public private partnership) and thus emphasized partnership between the two parties [26].

Enactment of the national higher education policy in 1999, among other things, set a direction towards increasing the number of trained health workers [29]. In order to achieve the latter, the number of students enrolled in science courses at the college and university levels was increased. This was made possible through the policy emphasis on increasing the number of science students enrolled in secondary school. However, due to the reduced budget in the education sector, as per the other social sectors following the SAP, the higher education policy called for the introduction of cost-sharing mechanisms in the education sector and the establishment of a legally created student loan scheme [29].

**Fig. 2:**
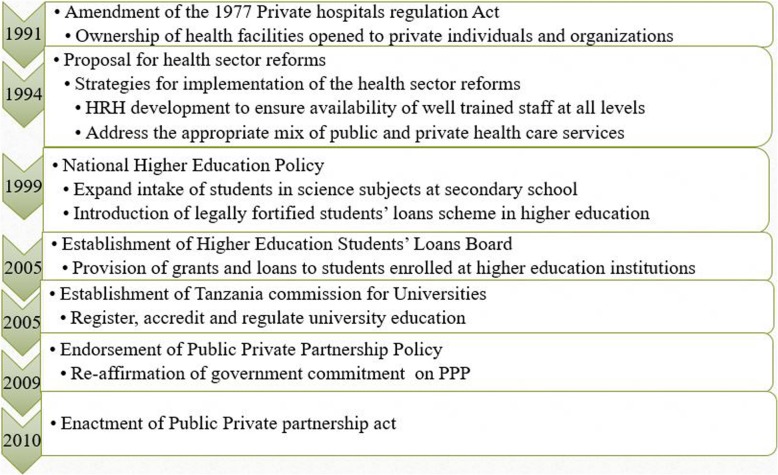
Implementation of public private partnership in the training of medical doctors after the 1990s’ health sector reforms in Tanzania

To fulfil this role, two independent organs—the Tanzania Commission for Universities (TCU) and the Higher Education Students’ Loans Board (HESLB)—were established under the Ministry of Higher Education, Science and Technology (MHEST). HESLB was established by Act No. 9 of 2004 and became operational in July 2005 [30, 31]. The HESLB was tasked to assist, on a loan basis, needy students who have secured admission to accredited higher learning institutions, but they have no financial capability to pay for their education [31]. TCU was established in 2005 to succeed the Higher Education Accreditation Council which was established in 1995 [32]. TCU was assigned the roles of accrediting institutions and university programmes, registering institutions, and regulatory functions in both the public and private sectors [32]. Figure 2 summarizes the implementation of PPP in the training of medical doctors after the 1990s’ health sector reforms.

In 2009, the government endorsed the first national public private partnership policy [33] which was followed by the enactment of a PPP act in 2010 [34].

#### Implementation of PPP and its outcomes on the training of doctors in Tanzania

From the interviews and document review, we found that implementation of PPP in the training of doctors in Tanzania has resulted into both intended and unintended outcomes.

### Intended outcomes

From the interviews and document review, we found that an increase in the number of medical training institutions, the use of public health facilities in the training of medical students by private universities, growth of collaboration among training institutions in the public and private sectors, government sponsorship of medical students in private training institutions, and the control of cost escalation in private training institutions by the government are among the positive outcomes linked to the implementation of PPP.

#### Increase in number of medical training institutions and number of graduates

From our review of the documents, we found that the number of medical training institutions increased from one in the early 1990s that was capable of enrolling less than 50 medical students annually to 11 which enrol more than 1000 medical students in 2015 (Table 3). However, despite the large number of private medical schools, the public medical school became the major producer of medical graduates until 2010 when it had produced over 60% of all medical doctors in the country [35]. Starting from 2010, the private training institutions combined started to produce almost double the number of medical doctors produced by the public institution annually [35].

**Table 3:** Medical training institutions in Tanzania in 2015 (Source: TCU 2015)

Name of institution	Ownership	Year
Muhimbili University of Health and Allied Sciences (MUHAS)^a^	Public	1963
International Medical and Technological University (IMTU)^a^	Private for-profit	1995
Hubert Kairuki Memorial University (HKMU)^a^	Private for-profit	1997
Kilimanjaro Christian Medical University College (KCMUCo)^a^	Private not-for-profit	1997
Catholic University of Health and Allied Sciences (CUHAS) ^a^	Private not-for-profit	2003
University of Dodoma (UDOM)	Public	2007
St. Francis University College for Health and Allied Sciences (SFUCHAS)^a^	Private not-for-profit	2010
Archbishop James University College (AJUCO)	Private not-for-profit	2011
St. Joseph University College of Allied and Health Science (SJCAHS)	Private not-for-profit	2011
Kampala International University Dar es Salaam (KIUD)	Private for-profit	2013
University of Dar es Salaam (UDSM) – School of Health Sciences	Public	2015

^a^By 2015, had produced at least one cohort of MD graduates

#### Supporting the training of medical students in private universities by the public health facilities

Informants from the private institutions stated that they receive training support from the public health facilities through signed memorandum of understandings to use those facilities for clinical trainings. They added that the arrangement has helped the private universities to deal with the challenges of limited space and patients for teaching as it is easier to access patients with different conditions in public hospitals than in private hospitals.

Our teaching hospital is small and, being a private hospital, we have a limited number and type of patients … . Sometimes it is difficult to access patients, as they are private … so we have memorandums of understanding with nearby public hospitals where we take our students for clinical rotations. (KI-1—private training institution)

#### Growth of collaboration among training institutions in public and private sectors

The participants stated that during the implementation of PPP for training, trainers in the public and private sectors meet regularly through the Committee of Vice Chancellors, Provosts and Principals in Tanzania (CVCPT) to discuss issues pertaining to training. From the review of documents, we found that the establishment of CVCPT was a requirement of the University Act No. 7 of 2005. However, CVCPT is composed of vice chancellors, provosts, and principals from all disciplines and from all universities in the public and private sectors, and it is not specific to health training institutions.

#### Government sponsorship of medical students to both public and private training institutions

From the document review and interviews, we found that the implementation of PPP in the training of doctors provided a forum for the government to support the training of doctors in private institutions through the provision of soft loans and grants. Informants from the private training institutions stated that the provision of loans and grants by the government has supported much of the growth of the private training institutions.

Now, we have more than 75% of students in first year that receive support from the government through the Higher Education Students’ Loans Board … . These fees help us to expand our capacity. (KI-3—private training institution)

#### Control of cost escalation in private training institutions

Many informants applauded the existence of a good regulatory framework in regulating training which helped to maintain training costs at an affordable rate, even at the private universities. They added that the tuition fees at all universities are discussed and agreed upon by many stakeholders and they come into effect after approval by the TCU. Informants from the government higher education regulatory bodies added that, before the establishment of the control mechanisms, some private institutions were charged very high tuition fees and they were payable using foreign currencies. They further added that the latter made even government support to the private training institutions to be difficult.

One of our main roles is to regulate the amount of tuition fees charged by the training institutions both in the public and private training institutions … .If you raise the fees without engagement of all key stakeholders and approval by TCU you are banned. (KI-10—MEST)

Some informants stated that the establishment of the central admission system (CAS) by the TCU provided greater economic benefit to parents and applicants. The latter was a result of paying application fees once and less for multiple applications to different universities instead of paying to individual institutions as it was before.

Now, you pay TZS 30,000 once for university application for up to eight different courses in up to five different training institutions. … Before Central Admission System, one had to pay varying amounts from TZS 20,000 to TZS 50,000 to different institutions. (KI-5—private training institution)

### Unintended outcomes

From the interviews, we also discovered that erosion of university autonomy and a perceived decline in the quality of medical training were some of the unintended outcomes of PPP in training.

#### Erosion of university autonomy

Most of the informants from the training institutions complained about the erosion of universities’ autonomy with regard to some decisions that are under their jurisdiction. They stated that the government, through its units, was making some decisions and implementing them or directing the universities to implement them, regardless of the fact that those decisions were mandated to the universities. For example, most informants complained that the government, without engaging the universities, selected and posted a large number of students to the universities in lieu of their capacities. Selection and admission of students are under the universities’ jurisdiction in consultation with the government.

Before the establishment of TCU, we were using WHO standards to determine the number of medical students … . TCU came up with its guidelines that set the faculty a student ratio of 1:15 and we adhered to it … . Unfortunately, when they started the central admission system, they took over the role of student selection without consulting us and they used this ratio to compute the number of students without knowing that not every faculty teaches every subject … . They then urged us to admit many students … . Now, in some courses, we have a ratio of 1:120. (KI-8—public training Institution)

#### Perceived decline in quality of medical training

Some informants perceived that the quality of medical training was declining from day-to-day. They stated that the admission of large numbers of students, exceeding the human and non-human resource capacity of the universities, was a major contributor. While some informants felt that some private institutions were happy due to the increased income from tuition fees when accepting large numbers of students, some informants from both public and private institutions complained that the increased number of students threatened the quality of training due to a critical shortage of resources. Cementing the complaints for quality, some informants stated that, the fact that the government was pushing admission of students to the training institutions without engaging the trainers, the standards of trainings were affected.

We plan our number of students based on the capacity of our teaching facilities, meaning the number of lecturers, size of the classes, capacity of our laboratory and the teaching hospital … . Sometimes it is a struggle when the government wants us to admit more than our capacity and we resist, knowing its impacts on the quality of the graduates. (KI-8—public training institution)

## Discussion

This policy analysis of PPP for the training of doctors following the 1990s’ health sector reforms in Tanzania provides insights into the context of the adoption of PPP, the actors involved, and how these influence one another, and the content and process of implementing PPP. The analysis also highlights the outcomes of PPP for the training of doctors.

### Tanzania at a crossroad: which way forward in addressing an increasing health workforce crisis?

Implementation of the reforms and, thus, the introduction of PPP in the training of doctors were not the favoured option in Tanzania; the country adopted it due to the worsening economic situation which manifested itself in the inability to meet the required budget and the mounting social demands of human resources for health [24]. As was the case in Tanzania, many low- and middle-income countries were compelled to undertake health sector reforms because of economic turbulence in their countries without adequate analysis of why, what, and how the reforms should be carried out [36, 37].

When reforms are driven by conjecture, implementation is prone to challenges either due to lack of understanding of the reforms itself, lack of resources to sustain the reforms, or due to the orthodox nature of institutions and individuals [38]. Although the implementation of PPP in the training of doctors in Tanzania was launched in the early 1990s, it was not until 2009—about two decades later—that a policy was adopted to define the government’s commitment to PPP [33].

In some other countries, although the government initiated the reforms, lack of resources to analyse what kind and extent of reforms were needed compelled these countries to request support from high-income countries. Malawi, for instance, initiated the health sector reforms by requesting that the United States Agency for International Development (USAID) fund and carry out an evaluation of health reform policy in Malawi [39]. Most of the high-income countries were practising market economies, and thus, the reforms they proposed were inclined towards market ethics which were not close to the mindsets of many leaders in low- and middle-income countries [37].

Leaving market forces to dictate the allocation and consumption of health care services, even with redefined roles of governments in funding social services, has been the subject of debate with regard to its effectiveness in ensuring accessibility and equity among the poor. For instance, it is often argued that leaving the health sector to market forces has contributed to the increased international brain drain of doctors from low- and middle-income countries to high-income countries [40, 41]. This, by itself, makes the low-income countries inject resources into supporting the training of doctors without reciprocal returns [8]. We argue that, unlike for other sectors, the government should regulate the labour market to minimize inequities that may arise due to market forces.

### Increasing numbers without addressing the root causes of the problem

Although our analysis revealed an increased number of training institutions from one to 11, and the number of MD graduates rose from less than 50 to about 1000 annually, the number of doctors employed both in the public and private health sector has not met the existing demand, particularly when the geographical aspect is factored in [35]. Despite the fact that some causes of the critical shortage of doctors in the 1980s have changed, most of the root causes have remained. Failure to offer employment in a fast-growing population and a growing health facilities network compounded by limited funding capacity to adequately remunerate the employed doctors have persisted, regardless of the introduction of PPP. In Tanzania, the public health sector constitute over 70% of the health sector; thus, majority of the graduates expect employment from the public sector. [35, 42–45]. Although it is possible to establish a private clinic after internship, the process is capital-intensive and it is not an easy readily available option to the newly graduates.

Furthermore, the rapid growth of the training institutions that enrol large numbers of medical students has changed the scenario from the absence of doctors in the market to a failure to employ the graduates [35, 46]. This seems to be partly due to a lack of linkage between the trainers and employers of the doctors, as revealed by our findings. Kohlen once argued that, with the existence of multiple stakeholders, there is a risk of fragmented coordination during the implementation process [47]. Although the aim of introducing PPP in the training of doctors was to increase their numbers, prompted by overwhelming shortages in the 1990s, the failure to plan their employment appropriately is largely responsible for the slow increase in the number of doctors in service provision [26, 35, 46]. This failure to absorb new graduates, just as in the 1980s, may be a stimulus for leaving the country in search of jobs if not addressed [46].

While it is a practice in many countries especially high-income ones to have common exit or examinations, or accumulation of professional credit points before licensing, in Tanzania, this is not the practice. Immediately after internship, the doctors are registered and licensed for practising medicine in the country. This is happening despite the increased number of students in the resource- constrained medical training institutions which also use different training curricula. It is high time for Tanzania to develop and implement a harmonized medical training curriculum and perhaps administer common exit examination before licensing medical doctors for practice. The latter will help to mitigate the rising concerns on the quality of graduates.

### Methodological consideration

The trustworthiness of findings in a qualitative study is demonstrated when such findings are worth believing [48]. To ensure the trustworthiness of our findings, we adopted the four criteria of Guba: credibility, dependability, transferability, and conformability [49]. The credibility of the findings of this study was enhanced through the triangulation of informant testimony with the experiences and rich information obtained from the study questions. In order to enhance the credibility and dependability of this study, triangulation of data collection techniques, study settings, and researchers was used. Data were collected using semi-structured interview guides and reviewing documents. In order to ensure that the findings reflected informants’ perspectives rather than the researchers’ understanding of the questions under study, all interviews were carried out in the natural settings of the informants; categories were inductively generated and presented with the support of subcategories and quotes. The transferability of the findings of this study is enhanced by the description of the study setting, context, data collection process, and analysis.

## Conclusion

From the findings of this study, PPP has attained its objective in terms of increasing the number of doctors graduating in the country because of increased number of training institutions and thus students’ enrolment. However, in the absence of harmonized training curricula and common exit exams, with the pressing critical shortage of faculty and training space in the training institutions; the increased number of training institutions and students enrolment challenge the quality of the graduates from these training institutions. Therefore, we suggest that low- and middle- income countries should revise their strategies of addressing the health workforce crisis in their countries by ensuring that whatever measures put in place, the quality of training should not be compromised.

## Abbreviations

AJUCO: Archbishop James University College
CAS: Central admission system
CUHAS: Catholic University of Health and Allied Sciences
HESLB: Higher Education Students’ Loans Board (HESLB)
HKMU: Hubert Kairuki Memorial University
IMF: International Monetary Fund
IMTU: International Medical and Technological University
KCMUCo: Kilimanjaro Christian Medical University College
KIUD: Kampala International University Dar es Salaam
LMICs: Low- and middle-income countries
MHEST: Ministry of Higher Education, Science and Technology (MHEST)
MUHAS: Muhimbili University of Health and Allied Sciences
PPP: Public private partnership
SAP: Structural adjustment programmes
SFUCHAS: St. Francis University College for Health and Allied Sciences
SJCAHS: Joseph University College of Allied and Health Science
TCU: Tanzania Commission for Universities
UDOM: University of Dodoma
UDSM: University of Dar es Salaam
WB: World Bank
WHO: World Health Organization
